# Detection of DNA Amplicons of Polymerase Chain Reaction Using Litmus Test

**DOI:** 10.1038/s41598-017-03009-z

**Published:** 2017-06-08

**Authors:** Dingran Chang, Kha Tram, Ben Li, Qian Feng, Zhifa Shen, Christine H. Lee, Bruno J. Salena, Yingfu Li

**Affiliations:** 1 Department of Biochemistry and Biomedical Sciences, McMaster University, 1280 Main St. W., Hamilton, ON L8S 4K1 Canada; 2 Department of Chemistry and Chemical Biology, McMaster University, 1280 Main St. W., Hamilton, ON L8S 4K1 Canada; 3 Department of Pathology and Molecular Medicine, McMaster University, 1280 Main St. W., Hamilton, ON L8S 4K1 Canada; 4 Department of Medicine, McMaster University, 1280 Main St. W., Hamilton, ON L8S 4K1 Canada; 5 Michael G. DeGroote Institute for Infectious Disease Research, McMaster University, 1280 Main St. W., Hamilton, ON L8S 4K1 Canada

## Abstract

We report on a new colorimetric DNA detection method that takes advantage of the power of polymerase chain reaction (PCR) and the simplicity of the classic litmus test. The strategy makes use of a modified set of primers for PCR to facilitate ensuing manipulations of resultant DNA amplicons: their tagging with urease and immobilization onto magnetic beads. The amplicon/urease-laden beads are then used to hydrolyze urea, resulting in the increase of pH that can be conveniently reported by a pH-sensitive dye. We have successfully applied this strategy for the detection of two hypervirulent strains of the bacterium *Clostridium difficile* that are responsible for the recent increase in the global incidence and severity of *C. difficile* infections. Furthermore, the viability of this test for diagnostic applications is demonstrated using clinically validated stool samples from *C. difficile* infected patients.

## Introduction

Polymerase chain reaction (PCR) is a popular DNA amplification technique and can create millions of amplicons of a target sequence in a short period of time^1–4^. This technique has been widely utilized for a variety of applications, including the detection of pathogenic bacteria^5–8^. PCR-based DNA detection is attractive for bacterial detection simply because specific DNA sequences can serve as reliable bacterial biomarkers and the amplification power of PCR permits the detection of a small number of bacteria before they can grow to infectious quantities^5–8^.

Although PCR has become a widely adopted technique in clinical laboratories, it has not become commonly used point-of-care or field tools. One significant roadblock that prevents such applications is the need of thermal cyclers that are often too expensive and bulky. However, there have been significant efforts towards miniaturizing PCR machines^9–12^. Another significant barrier that restricts PCR from becoming a popular field tool is the lack of simple yet effective signal transduction mechanisms that permit detection of PCR products without the use of expensive equipment (such as real-time PCR machine) and complicated process (such as DNA separation by gel electrophoresis). These issues reduce the utility of PCR as field tools. One approach to address this problem is to develop simple colorimetric assays that can be easily implemented in the field to detect DNA amplicons of PCR. Recently, our group reported an approach of adopting the classic litmus test for bacterial detection using a protein-activated DNAzyme and the protein enzyme urease^13^. Herein we describe a strategy for adopting the same litmus test for the detection of PCR amplicons through the use of a set of specially modified DNA primers and urease.

**Figure 1:**
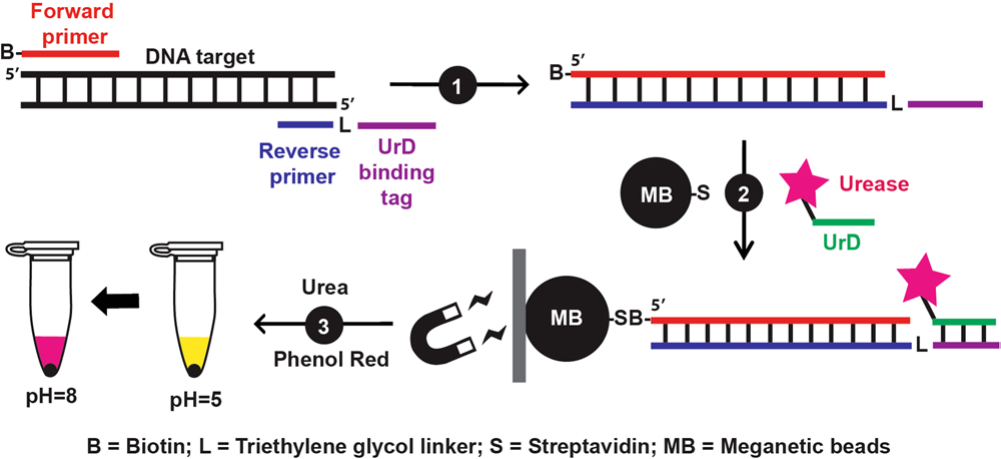
Assay principle. (1) PCR reaction. (2) Immobilization of PCR product and urease labeled DNA onto magnetic beads. (3) Litmus test.

Our devised strategy is illustrated in Fig. 1. Two modified DNA primers are used to carry out PCR. The forward primer contains a 5′-biotin and the reverse primer contains a triethylene glycol linker that separates the target binding sequence (blue) from the sequence (purple) that is designed to hybridize with the DNA strand (green) coupled onto urease, a conjugate denoted “UrD” in this report. By this design, the PCR amplicons can be immobilized on the surface of magnetic beads containing streptavidin and the immobilized amplicons are capable of capturing UrD. The amplicon/urease-charged beads can then be used to hydrolyze urea, resulting in the increase of pH that can be reported by a pH-sensitive dye, such as phenol red, which can produce a sharp, yellow-to-pink transition when the solution pH changes from acidic to basic. Simply put, the proposed strategy converts the detection of DNA into tracking of pH increases using procedures that are easy to carry out.

To validate the proposed idea, we chose to develop a test that can be used to detect two hypervirulent strains of *Clostridium difficile* (*C. difficile*) known as 027/NAP1 and 078/NAP7. *C. difficile* is a gram-positive, spore-forming anaerobic bacterium that has been identified as the leading cause of diarrhea in developed countries and pseudomembranous colitis in humans^14^. The incidence and mortality of *C. difficile* infection (CDI) has increased dramatically over the past 15 years and CDI has become a common problem in hospitals across North America, Europe and some regions of Asia^15, 16^. This has been mostly attributed to the emergence of highly virulent strains. One such strain was characterized as ribotype 027, as North America Pulsed-field type NAP1 by pulsed field gel electrophoresis (PFGE) and was subsequently referred as 027/NAP1^17^. The 027/NAP1 strain is known for its high-level resistance to fluoroquinolones, with markedly high toxin production and a mortality rate three times as high as that associated with less virulent strains, such as the ribotype 001^17–20^. Another epidemic strain of *C. difficile*, known as 078/NAP7, has also been described as hypervirulent since it can cause symptoms of similar severity to strain 027/NAP1. However, 078/NAP7 affects the younger population and is more frequently community-associated^21–24^. Identification of these epidemic strains may assist in determining relatedness of strains and recognizing transmission of *C. difficile* within healthcare facilities.

Current methods available for the diagnosis of CDI include cytotoxicity assays, anaerobic toxigenic culture, enzyme immunoassay (EIA) and nucleic acid amplification-based tests^16, 25^. These methods generally target toxins or their associated toxin genes and are unable to discriminate epidemic strains from non-epidemic strains^14, 25–27^. Meanwhile, current strain typing methods for *C. difficile*, such as ribotyping, restriction endonuclease analysis and PFGE, require culture of the organism, lack discriminatory power and are performed in reference laboratories, with resulting delays of days to weeks^28, 29^. Consequently, there is a significant need for a simple and rapid method for identifying epidemic *C. difficile* strains.

The virulence of *C. difficile* is mainly caused by toxin A and toxin B which are encoded by *tcdA* and *tcdB*, respectively, and their expression is regulated by *tcdR* (positive regulator) and *tcdC* (negative regulator)^30–32^. Interestingly, mutations in *tcdC* were found in both strains 027/NAP1 and 078/NAP7. A single-base deletion at nucleotide position 117 of *tcdC* (*∆1stop-tcdC*) has been found in the strain 027/NAP1 and this single nucleotide deletion results in a frame shift that introduces a stop codon at nucleotide position 196^33, 34^. Consequently, this generates a shortened protein of 65 residues. Similarly, strain 078/NAP7 carries a *tcdC* with a C184T transition (*TAAstop-tcdC*) leading to a truncated protein of 61 residues^35^. These mutations in *tcdC* have been proposed as a possible explanation for the increased virulence of the epidemic strains. Meanwhile, since these mutations are well conserved in strains 027/NAP1 and 078/NAP7, they can serve as molecular markers for the rapid identification of the hypervirulent *C. difficile* strains^34–36^.

It is well established that PCR can achieve highly specific DNA detection. For example, allele-specific PCR has been developed for Single Nucleotide Polymorphisms (SNP) detection using a DNA primer containing a single mismatch at 3′-end. This principle was used in this study to achieve the detection of mutations in t*cdC* genes associated with 027/NAP1 and 078/NAP7.

**Figure 2:**
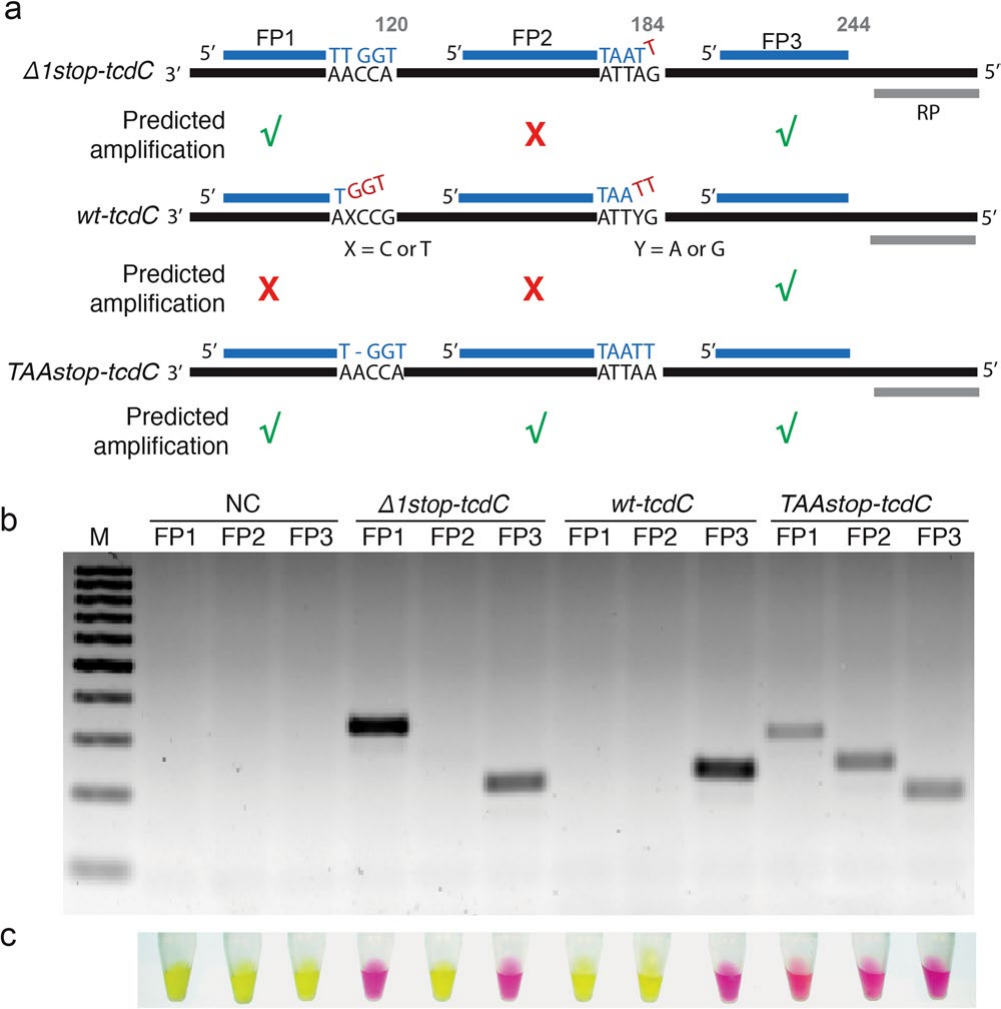
PCR-based tests for the wildtype and two mutated *tcdC* genes. (**a**) Primer design. Primers for *tcdC* genes are color-coded. The mismatched 3′ terminus between forward primer and template are shown in red. Green tick and red cross denote a successful and unsuccessful PCR reaction, respectively. (**b**) Analysis of PCR products by 2% agarose gel electrophoresis. (**c**) Litmus test with PCR products. The photographs were taken at 30 minutes.

Through sequence analysis of *tcdC* (Figure S1, Supplementary Information), three forward primers (FP1, FP2 and FP3) and one reverse primer (RP) were designed for the discrimination between four possible outcomes: wild type *tcdC* (*wt-tcdC*), *∆1stop-tcdC*, *TAAstop-tcdC* and no *tcdC*, as illustrated in Fig. 2a (sequences of the DNA molecules used for this work are provided as Table S1 in the Supplementary Information). We found that both *∆1stop-tcdC* and *TAAstop-tcdC* have a thymine at position 120, whereas *wt-tcdC* has a cytosine at this position. P1 with 3′-thiamine was then designed to detect this difference. Similarly, P2 targets adenine at position 184 of *tcdC* to discriminate *TAAstop-tcdC* and *∆1stop-tcdC*. P3 was designed to amplify all *tcdC* variants and could be used to identify the presence of *tcdC* gene and play the role of quality control during PCR. Since P1, P2 and P3 have similar T_m_, they should theoretically have comparable PCR yields under same condition. The design of each primer was checked by BLAST to avoid nonspecific amplifications. Secondary structures and self-pairing of these primers were also examined.

Three *C. difficile* strains, 027/NAP1 (with *∆1stop-tcdC*), 078/NAP7 (with *TAAstop-tcdC*) and 001/NAP2 (with *wt-tcdC*) were tested to examine the performance of the designed primers. Genomic DNA from each strain was extracted based on a previous described method. 200 ng of genomic DNA was used as starting material for 28 cycles of PCR amplification. Agarose gel analysis indicates that a significant amount of PCR product was generated when primers matched the template, whereas no PCR product was observed in the absence of target gene or when using primers with mismatched 3′ termini (Fig. 2b). Based on the performances of three primer pairs toward each strain, we could successfully discriminate between epidemic *C. difficile* strains, 027/NAP1 and 078/NAP7 and non-epidemic *C. difficile* strain 001/NAP2. We also tested the performance of each primer set by changing the number of PCR cycles. Figure S2 indicates that non-specific products were generated after more than 30 cycles of PCR. This is consistent with previous findings that primer 3′ nucleotide mismatches could reduce PCR efficiency but not fully inhibit the reaction^26^. As a result, we proceeded to perform each assay within 30 cycles of PCR.

To achieve colorimetric detection of PCR products, we firstly conjugated urease with an oligonucleotide as reported in a previous method (see details in the methods)^13^. This conjugated urease-DNA (UrD) is used to hybridize to the extended binding region found in the reverse primer. Although the UrD does not contain a biotin modifier, the UrD has a tendency to non-specifically bind to magnetic beads at high concentrations and increase the background signal. To circumvent non-specific binding, varying concentrations of UrD was incubated with magnetic beads and tested with addition of phenol red (pH indicator) and urea after thorough washing of the beads. From this control experiment, we can determine the suitably diluted UrD solution to be used in subsequent experiments. As presented in Figure S3, we found that there was no observed color change after 2 hours, when 1 μL of 0.6 μM UrD in 10 μL of magnetic beads was used. Therefore, we chose to use this stock concentration for further assay development and testing.

We then carried out the colorimetric test on the PCR sample, expecting that the presence of PCR products would induce a color change. PCR positive products indeed yielded a color change from yellow to pink while samples that remained yellow indicated the absence of amplified PCR products and therefore did not capture the UrD. As shown in Fig. 2c, the results of three colorimetric reactions for each strain form a triplet pattern (no color change for absence of *tcdC*, one color change for *wt-tcdC* (non-epidemic strain), two color changes for *∆1stop-tcdC* (epidemic strain 027/NAP1), and three color changes for *TAA stop-tcdC* (epidemic strain 078/NAP7)). These patterns can then be used to identify epidemic *C. difficile* strains.

**Figure 3:**
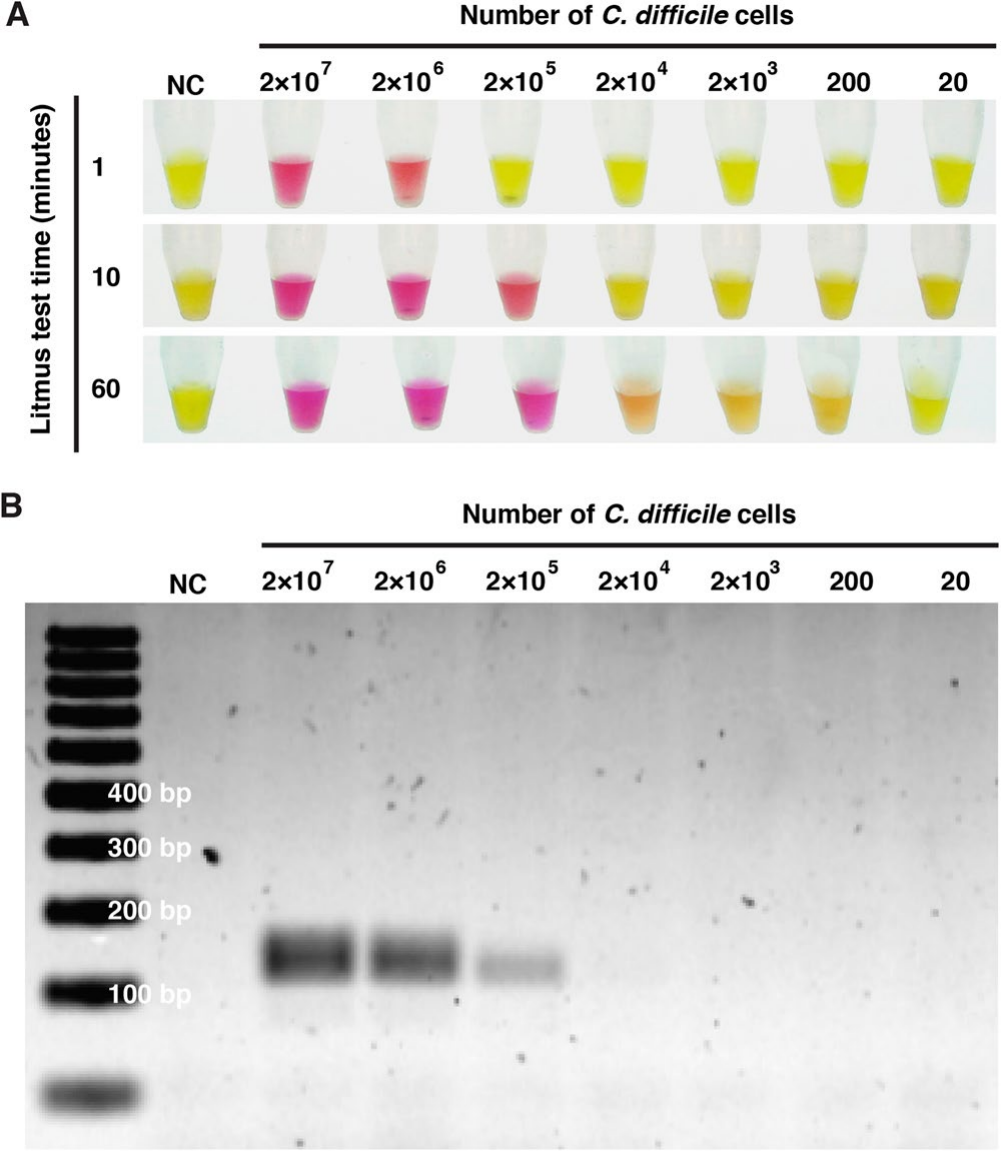
Sensitivity of PCR-litmus test for *C. difficile* detection. 28 cycles of PCR was performed with genome DNA prepared from various numbers of *C. difficile* cells. Strain ATCC 1803, forward primer P3 and reverse primer RP were used in this test. (**A**) Litmus test with PCR products. The photograph was taken after a signal-producing time of 1 minute (top panel), 10 minutes (middle) and 60 minutes (bottom). (**B**) Analysis of PCR products by 2% agarose gel electrophoresis.

The sensitivity of the PCR-litmus assay was evaluated by testing genomic DNA isolated from serially diluted *C. difficile* stocks containing a known number of cells (Fig. 3a). A sharp color transition was observed for the sample containing 2 × 10^6^ cells after incubation for 1 minute (top panel) and 2 × 10^5^ cells after incubation for 10 minutes (middle panel). A subtle but detectable color transition, in comparison to the reference samples (without target), was observed for the sample containing 200 cells after color development of 1 hour. We compared the colorimetric test to conventional agarose gel based tests where PCR products were visualized through the staining of DNA-binding dyes. Three commonly used dyes were chosen for this experiment (SYBRSafe ; Fig. 3b), SYBRGold and GelRed (Figure S4). We found the dye-staining methods resulted a detection limit of approximately 10^4^–10^5^ bacteria cells. Therefore our litmus test exhibits a better detection sensitivity by as much as 100 fold. We have also performed spectroscopic analysis to quantify the detection sensitivity by plotting OD570/OD443 vs. cell numbers (Figure S5), based on the fact that the color of phenol red exhibits a gradual transition from yellow (λmax = 443 nm) to red (λmax = 570 nm) when the pH of a test solution changes from acidic to basic^37^. This analysis confirms that our method can indeed detect as low as 200 cells; however, the spectroscopic analysis did not lead to improved detection sensitivity.

**Figure 4:**
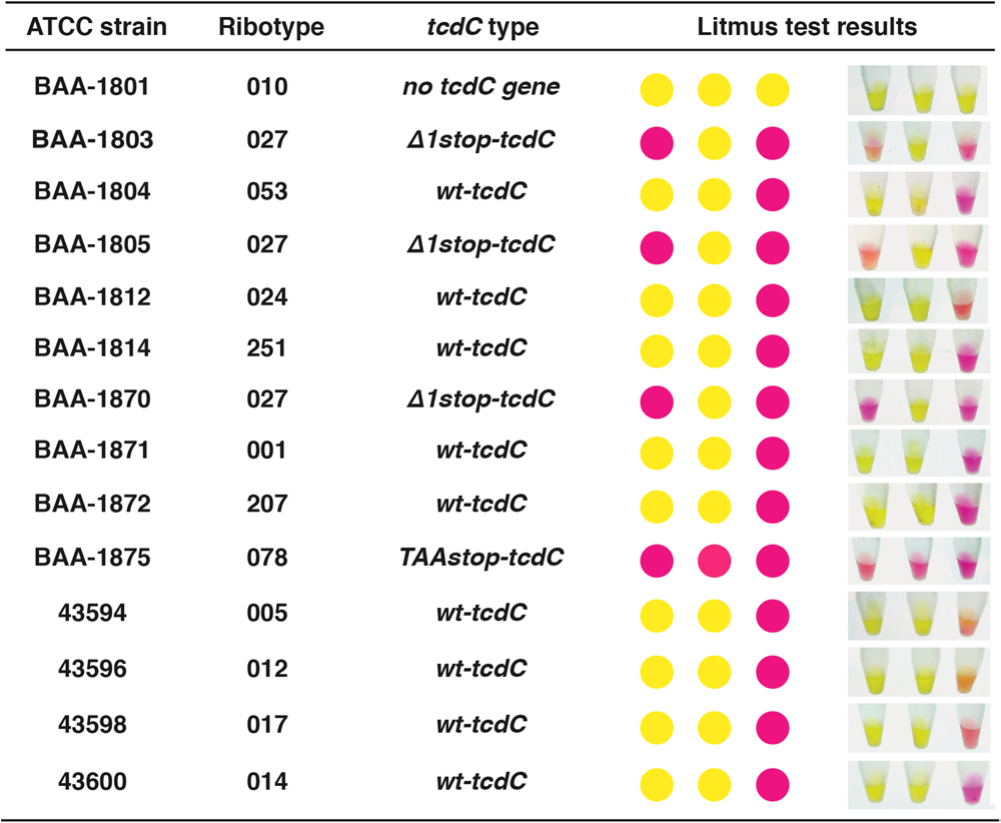
PCR-litmus test with genomic DNA from 14 different *C. difficile* strains. The *tcdC* genes from all the strains were sequenced and their nucleotide sequences were compared with database entries by using PubMLST program. Ribotype of each strain is provided by ATCC. The photographs of test were taken at 30 minutes.

To further verify the accuracy of the assay in strain discrimination, we applied the assay towards genomic DNA extracted from 14 different strains of *C. difficile* (see Fig. 4). In addition, the *tcdC* genes from all these strains were independently sequenced to confirm their identity. It was found that our PCR-litmus test accurately identified three 027/NAP1 strains and one 078/NAP7 strain of *C. difficile* in reference to the *tcdC* sequencing analysis and the documentation provided by ATCC.

**Figure 5:**
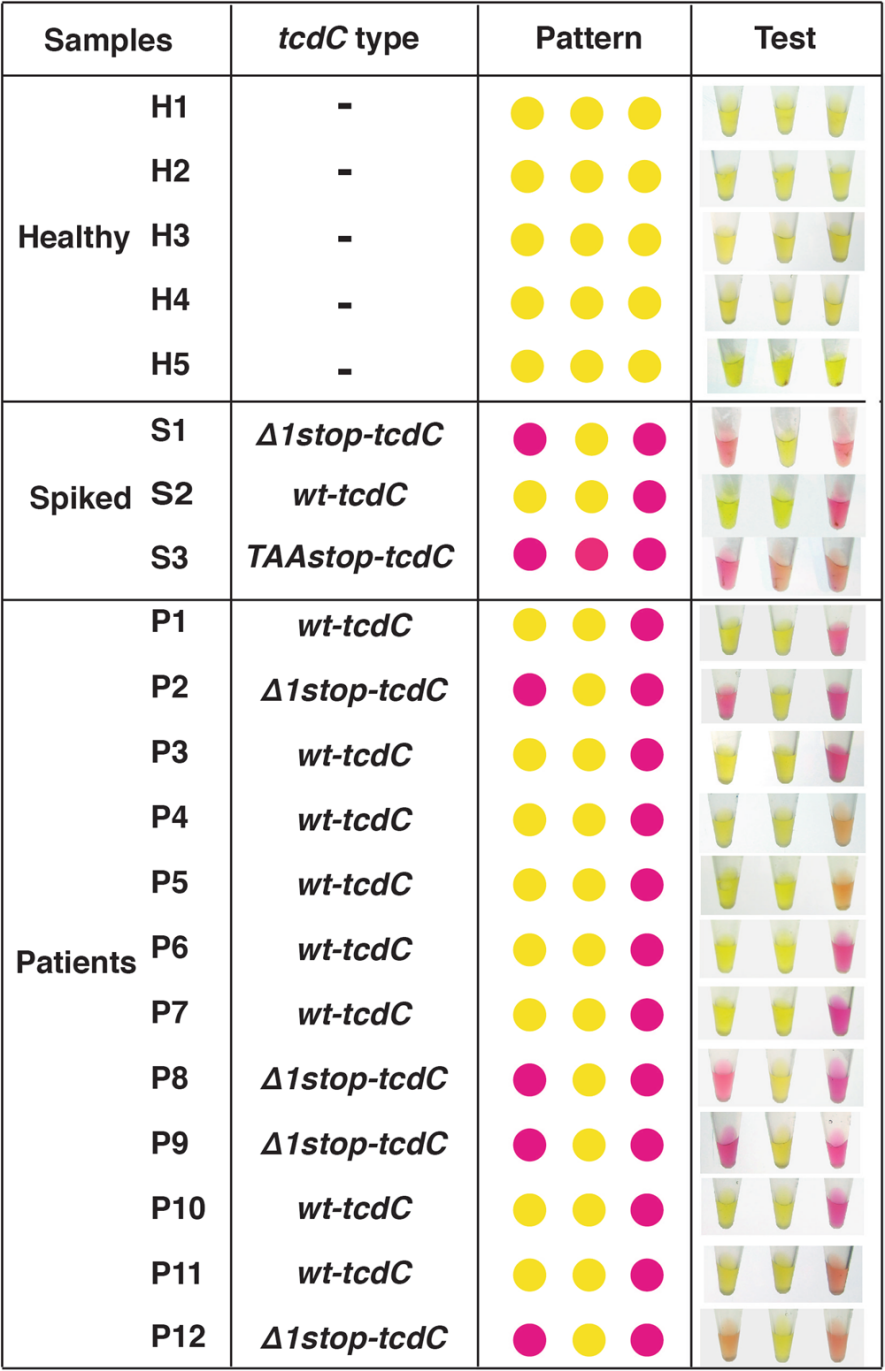
PCR-litmus test with DNA from stool samples. The *tcdC* gene information was acquired by DNA sequencing. The photographs of test were taken at 30 minutes.

Evidence of a *C. difficile* infection can be determined non-invasively by analyzing a patient’s stool sample. However, due to the composition and complexity of these samples, we next investigated the utility of our PCR-litmus assay in clinically relevant settings. We randomly selected stool samples of 12 CDI patients and 5 healthy donors obtained from St. Joseph’s Healthcare (Hamilton, Canada). In addition, healthy stool samples were also spiked with known strains of *C. difficile* cells with a concentration of 10^8^ cells/g. Total DNA from each sample was extracted using Powerfecal DNA isolate kit (MO BIO LABORATORIES, USA). 100 ng of total DNA from each sample was used for PCR amplification. From Fig. 5, stool collected from healthy patients did not indicate color change. All 3 spiked samples revealed color patterns indicative of their respective spike strains. Of the 12 CDI patient samples, 4 presented a pattern of 027/NAP1 while the other 8 were identified as *C. difficile* (with *wt-tcdC*) but neither as 027/NAP1 nor 078/NAP7. These patterns are consistent with the *tcdC* sequencing results. These tests demonstrated that our approach could potentially be used for clinical diagnosis.

In summary, we have developed a simple method that can achieve colorimetric detection of DNA amplicons. To the best of our knowledge, our work represents the first example of adopting the classic litmus test for PCR-based DNA detection through the use of DNA conjugated urease. We have also shown that this method can be used to identify epidemic strains of *C. difficile* through the use of primers that can selectively amplify the *tcdC* genes in these strains. We have further illustrated the clinical usefulness of the proposed method by identifying epidemic strains of *C. difficile* in stool samples.

The featured PCR-litmus test can be easily extended to the detection of other gene targets simply by changing the primers, and thus we envision that this method can find wide applications in diverse fields.

## Methods

### Materials

All DNA oligonucleotides were prepared by automated DNA synthesis using standard phosphoramidite chemistry (Integrated DNA Technologies, Coralville, IA, USA) and purified by 10% denaturing (8 M urea) polyacrylamide gel electrophoresis (dPAGE). Their concentrations were determined spectroscopically. Deoxynucleoside 5′-triphosphates (dNTPs), SYBR Safe and SYBR Gold dyes were purchased from Thermo Scientific (Ottawa, ON, Canada). GelRed was obtained from Bitium (Fremont, CA, USA). Thermus thermophilus DNA polymerase was acquired from Biotools. Streptavidin coated magnetic beads of 1.5 μm (BioMag-SA) was purchased from Bangs Laboratories Inc. Urease powder from Canavaliaensiformis (Jack bean), maleimidobenzoic acid N-hydroxy-succinimide ester (MBS), phenol red were obtained from Sigma-Aldrich. Water was purified with a Milli-Q Synthesis A10 water purification system. All other chemicals were purchased from Bioshop Canada and used without further purification.

### Bacterial strains and routine culture conditions

A panel of 14 *C. difficile* strains obtained from the American Type Culture Collection (ATCC; Manassas, USA) was used in this study. Cells were cultured in cooked meat broth medium (Sigma-Aldrich) under anaerobic condition with gas mixture of 80% N_2_, 10% CO_2_, 10% H_2_ in a Whitley Anaerobic Workstation (Don Whitley Scientific).

### Total DNA extraction from bacteria strains

Cells were grown in 5 mL of cooked meat broth medium until OD_600_ reached ~1. 200 μL of cultures were spun down (11000 g, 5 min) in order to remove the culture medium; and the obtained pellets were suspended in 200 μL of 5% Chelex 100 (Bio-Rad) with 0.2 mg protease K. The mixture was then vortexed and incubated at 56 °C for 30 min and then 95 °C for 15 min. After centrifugation for 10 min at 10 000 g, the supernatant was transferred into a fresh tube and stored at 4  °C until PCR testing.

### PCR primer design

For tcdC gene, primer design was conducted with the OligoAnalyzer 3.1(http://www.idtdna.com/calc/analyzer) after alignment of 26 tcdC_fragment gene sequences got from PubMLST (http://pubmlst.org/). All primers were checked using the alignments of sequences, and subsequently with the basic local alignment search tool (BLAST; http://www.ncbi.nlm.nih.gov/BLAST/). Secondary structures and self-pairing of all primers were also checked with the OligoAnalyzer 3.1. All primers are listed in Table S1.

### PCR reaction

The PCR mixture (50 µL) contained 200 ng of DNA, 0.5 µM of forward primer (P1, P2 or P3) and reverse primer (RP), 200 µM of dNTPs (dATP, dCTP, dGTP and dTTP), 1 × PCR buffer (75 mM Tris-HCl, pH 9.0, 2 mM MgCl_2_, 50 mM KCl, 20 mM (NH_4_)_2_SO_4_) and 1 units of Thermus thermophilus (Tth) DNA polymerase. The DNA was amplified using the following thermocycling steps: 94 °C for 5 min; 28 cycles of 94 °C for 1 min, 60 °C for 1 min and 72 °C for 1 min; 72 °C for 3 min.

### DNA-urease conjugation

Urease-DNA was prepared according to a previously reported method^29^. An MBS solution (6.4 mM) was made by dissolving 2 mg MBS (6.4 μmol) in 1 mL of dimethyl sulphoxide (DMSO). Similarly a urease solution was produced by dissolving 1.5 mg urease (2.75 nmol urease haxamer) powder in 1 mL of 1 × PBS buffer (pH 7.2). 10 nmol NH_2_-DNA and 3.2 μL of the MBS solution (20 nmol) were mixed and adjusted to a final reaction volume of 400 μL with 1 × PBS buffer, and allowed to react at room temperature. After 30 min, the mixture was passed through a membrane based molecular sizing centrifugal column with a molecular weight cut-off of 3,000 Daltons (NANOSEP OMEGA, Pall Incorporation) in order to remove excess MBS. The column was washed with 50 μL of 1 × PBS buffer 3 times and the DNA was resuspended in 100 μL of 1 × PBS buffer. The urease solution (1 mL,  2.75 nmol) was then added to the MBS activated DNA. The conjugation reaction was allowed to proceed at room temperature for 1 h. The mixture was filtered through a 300,000-Dalton cut-off centrifugal column. The DNA-urease conjugate (Ur-DNA) was then washed with 50 μL of 1 × PBS buffer 3 times, and resuspended in 160 μL of 1 × PBS buffer. The ratio of DNA over urease in UrD was found to be 1.54 using a previously published method^38^. Details are provided as the Supplementary Methods in Supplementary Information.

### Litmus test

50 μL of the above PCR reaction mixture was incubated with 50 μL of binding buffer (10 mM Tris-HCl, pH 7.5, 3 M NaCl, 1 mM MgCl_2_, 0.01% tween 20) along with 10 μL of magnetic beads (MB) for 15 minutes. Then it was placed in a magnet holder to separate the supernatant and MB. Then the MB was suspended in 100 μL of binding buffer with 1 μL of 0.6 μM UrDNA (1 pmol). After 15 min of incubation, MB was washed with 100 μL of binding buffer four times and then resuspended in 70 μL of acetic acid buffer (0.1 mM, pH 5). Then 10 μL of 0.04% phenol red and 100 μL of substrate solution (3 M NaCl, 60 mM MgCl_2_, 50 mM urea) were added. Note that this substrate solution should have a starting pH of 5.0. A photograph was taken after a signal-producing time of 0–1 h according to individual experiments for Figs 2, 3, 4 and 5.

### Sequencing of *tcd*C genes

Each *tcdC* gene was amplified by PCR as previously described^33^. The reaction mixture contained 1 × PCR buffer, 200 pmol of each dNTPs, 25 pmol of the FP-F and RP-F (Table S1), and 1 U of Tth DNA polymerase. The template was denatured for 5 min at 94 °C, and DNA was amplified for 30 cycles consisting of 1 min at 94 °C, 1 min at 50 °C, and 1 min at 72 °C. The PCR products were sequenced at the Mobix Lab (McMaster University). The nucleotide sequences were compared with database entries by using the PubMLST program.

### Sensitivity test

A single colony of strain ATCC1803 from an anaerobic cooked meat broth agar plate was taken and cultured in 5 mL of cooked meat broth medium overnight. The bacterial culture was then diluted in 10-fold intervals seven times with cooked meat broth medium; 100 μL of 10^−5^, 10^−6^ and 10^−7^ dilutions were placed on a cooked meat broth plate and cultured for colony development in order to calculate the cell numbers (average colony-forming units) for each dilution. Meanwhile, 200 μL of each diluted cultures were used for DNA extraction and PCR-litmus test. After a signal producing time of 1 hour, measurements at 443 nm and 570 nm were performed by using a microplate scanning spectrometer (TECAN M1000). Agarose gel based tests were also performed. SYBR Safe, SYBR Gold and GelRed were used for gel staining.

### Stool sample test

Stool samples from 5 healthy donors and 12 patients infected by *C. difficile* were obtained from St. Joseph’s Healthcare-Hamilton, with informed consent from all the healthy donors and infected patients. The experimental protocols were approved by Hamilton Integrated Research Ethics Board (HiREB), McMaster University (Hamilton, Ontario, Canada). The methods were carried out in accordance with the relevant guidelines and regulations. The three spiked samples were also prepared by adding ~2 × 10^7^ *C. difficile* cells (ATCC 1870, ATCC1871 or ATCC1875) into 200 mg of stool sample from healthy donors. A Powerfecal DNA isolate kit (MO BIO LABORATORIES, USA) was used to extract total DNA from stool samples, and 100 ng of total DNA from each sample was used as template DNA for PCR-litmus test. The DNA was amplified using the following thermocycling steps: 94 °C for 5 min; 30 cycles of 94 °C for 1 min, 60 °C for 1 min and 72 °C for 1 min; 72 °C for 3 min.

### Data Availability

All data generated or analysed during this study are included in this published article and its Supplementary Information.

## Electronic supplementary material

Supplementary Information
