# Supplementary material for: Detection of DNA Amplicons of Polymerase Chain Reaction Using Litmus Test
Source: Sci Rep. 2017 Jun 8;7:3110. doi: 10.1038/s41598-017-03009-z (PMC5465217; doi:10.1038/s41598-017-03009-z)
Supplement: Supplementary file 1 — Supplementary Information [file 41598_2017_3009_MOESM1_ESM.pdf]

Detection of DNA Amplicons of Polymerase Chain Reaction Using Litmus Test

Dingran Chang[1], Kha Tram[2], Ben Li[1], Qian Feng[2], Zhifa Shen[1], Christine H. Lee[3], Bruno J. Salena[4] and Yingfu Li[1,2]*

[1]Department of Biochemistry and Biomedical Sciences, [2]Department of Chemistry and Chemical Biology, [3]Department of Pathology and Molecular Medicine, [4]Department of Medicine, McMaster University, 1280 Main St. W., Hamilton, ON L8S 4K1, Canada

*Email: liying@mcmaster.ca

Supplementary Information

SUPPLEMENTARY METHODS

Conjugate quantification. The average loading of DNA per urease can be calculated by measuring the absorbance at 260 and 280 nm. The concentration of the conjugate can be calculated as follows:[1]

$$A_{260UrDNA} = A_{260urease} + A_{260DNA} \quad (1)$$

$$A_{280UrDNA} = A_{280urease} + A_{280DNA} \quad (2)$$

$$A_{260DNA} / A_{280DNA} = \alpha \quad (3)$$

$$A_{260urease} / A_{280urease} = \beta \quad (4)$$

As shown in Figure S6, the ratio α and β of the absorbance at 260 and 280 nm of NH_2-DNA (α =1.72) and urease (β = 0.72) were determined by using Nanovue plus spectrophotometer. Insertion of Eqs. 3 and 4 into 1 and 2, respectively, leads to Eqs. 5 and 6

$$A_{260UrDNA} =\beta A_{280urease} + A_{260DNA} \quad (5)$$

$$A_{280UrDNA} = A_{280urease} + (A_{260DNA}\times 1/\alpha) \quad (6)$$

Insertion of Eqs.5 into 6, leads to

$$A_{260DNA} = (\alpha A_{260UrDNA} -\alpha\beta A_{280UrDNA})/(\alpha-\beta) \quad (7)$$

$$A_{280urease} = (\alpha A_{280UrDNA} -A_{260UrDNA})/(\alpha-\beta) \quad (8)$$

The absorbance of UrDNA was determined to be 0.278 at 260 nm and 0.235 at 280 nm. By insertion to Eqs. 7 and 8,

$$A_{260DNA} = (1.72 \times 0.278 -1.72 \times 0.72\times 0.235) / (1.72-0.72) = 0.187$$

$$A_{280urease} = (1.72 \times 0.235 - 0.278) / (1.72-0.72) = 0.126$$

Then, according to Lambert-Beers-Law: $c = A/(\varepsilon\times L)$, we can get

$$c_{DNA} = A_{260DNA}/(\varepsilon_{DNA}\times L) \quad (9)$$

$$c_{urease} = A_{280urease}/(\varepsilon_{urease} \times L) \quad (10)$$

According to OligoAnalyzer 3.1 (http://www.idtdna.com/calc/analyzer), ε_{260} of NH_2-DNA is 316,900 $M^{-1}\cdot cm^{-1}$. Based on previous study[2], ε_{280} of Jackbean urease monomer is 54,780 $M^{-1}\cdot cm^{-1}$. Here, L is 0.05 cm when using Nanovue plus spectrophotometer. Taking into Eqs. 9 and 10,

$$c_{DNA} = 0.187 / (316900\ M^{-1}\cdot cm^{-1}\times 0.05\ cm) = 11.8\ \mu M$$

$$c_{urease} = 0.126 / (54780\ M^{-1}\cdot cm^{-1} \times 0.05\ cm) = 46.0\ \mu M$$

Based on above calculations, the Ur-DNA concentration was 11.8 µM of DNA equals to about 46.0 µM of urease monomer. If ureases are in the form of monomers, the DNA/urease ratio will be 0.26.

However, Jackbean ureases are known to exist in the form of homohexamers, the DNA/urease ratio will be 1.54.

1. Kukolka, F., Lovrinovic, M., Wacker, R. & Niemeyer, C. M. Covalent coupling of DNA oligonucleotides and streptavidin. *Methods Mol. Biol.* **283,** 181–96 (2004).
2. Real-Guerra, R., Carlini, C. R. & Stanisçuaski, F. Role of lysine and acidic amino acid residues on the insecticidal activity of Jackbean urease. *Toxicon* **71,** 76–83 (2013).

SUPPLEMENTARY TABLES AND FIGURES

Table S1. Sequences used in PCR-litmus test.

Name	Sequences (5′-3′)
FP1	Bio-CTCTACTGGCAT TTATTT TGGT
FP2	Bio-CATGAGGAG GTCATTTCTAATT
FP3	Bio-AGAAACTTTAAATAGCAAATTGTCTG
RP	CTTGACACTATTACGCACGCTTCACTATTTTTTTTT<br>T-iSp9-TACCAGTATCATATCCTTTCTTCTC
NH_2-DNA	AmMC6T- TTTTTTAGTGAAGCGTGCGTAATAGTG<br>TCAAG
FP-F	TTAATTAATTTTCTCTACAGCTATCC
RP-F	TCTAATAAAAGGGAGATTGTATTATG

Note: "Bio" represents biotin, "AmMC6T" represents amino modifier C6, and "iSp9" in the middle of the sequence is a triethylene glycol linker to stop extension of polymerase.

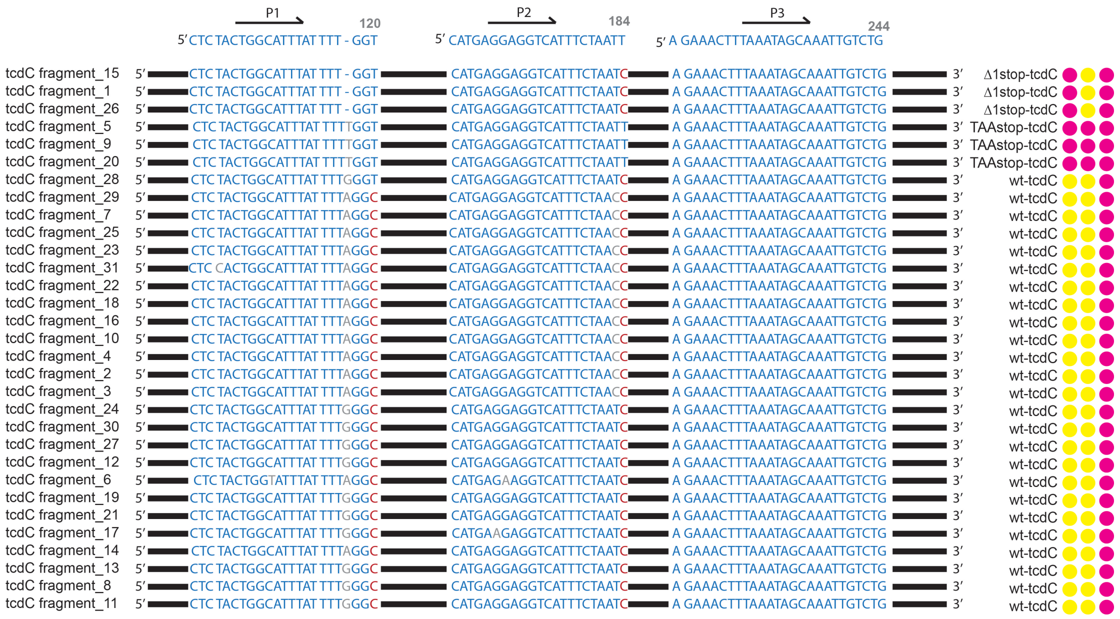

Figure S1. Sequence alignment and primer design for identification of epidemic strains of *C. difficile*. An alignment of 31 *tcdC*_fragment gene sequences (PubMLST http://pubmlst.org/) (left panel) and corresponsive triplet patterns generated by PCR-litmus test (right panel) are shown. The primer binding regions are color-coded. The mismatched 3′ terminus between forward primer and template are shown in red.

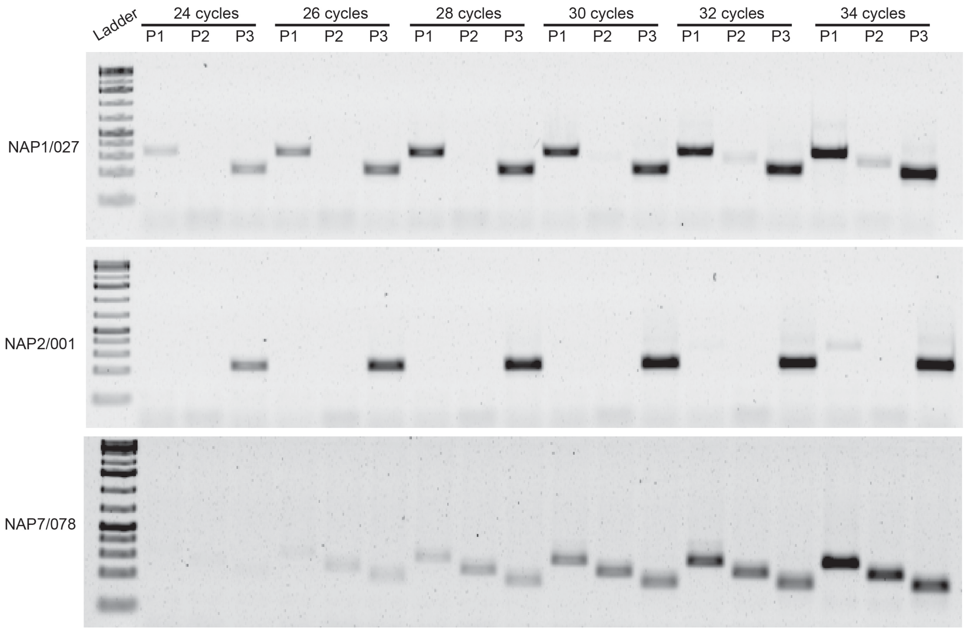

Figure S2. Evaluation of the effect of PCR cycles on primer specificity. 200 ng of genomic DNA prepared from strain 027/NAP1, 001/NAP2 and 078/NAP7 were used as starting material. PCR products are analyzed by 2% agarose gel electrophoresis.

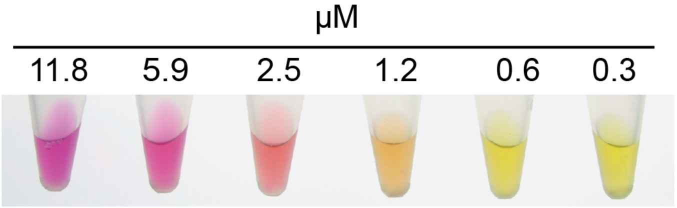

Figure S3. Optimization of concentration of UrD in litmus test. Different concentrations of UrD were incubated with binding buffer along with 10 µL of MB for 15 minutes. MB was then washed four times with 100 µL of binding buffer and resuspended in 70 µL of acetic acid buffer. This was followed by addition of 10 µL of 0.04% phenol red and 100 µL of substrate solution. The photograph was taken after a signal-producing time of 2 h.

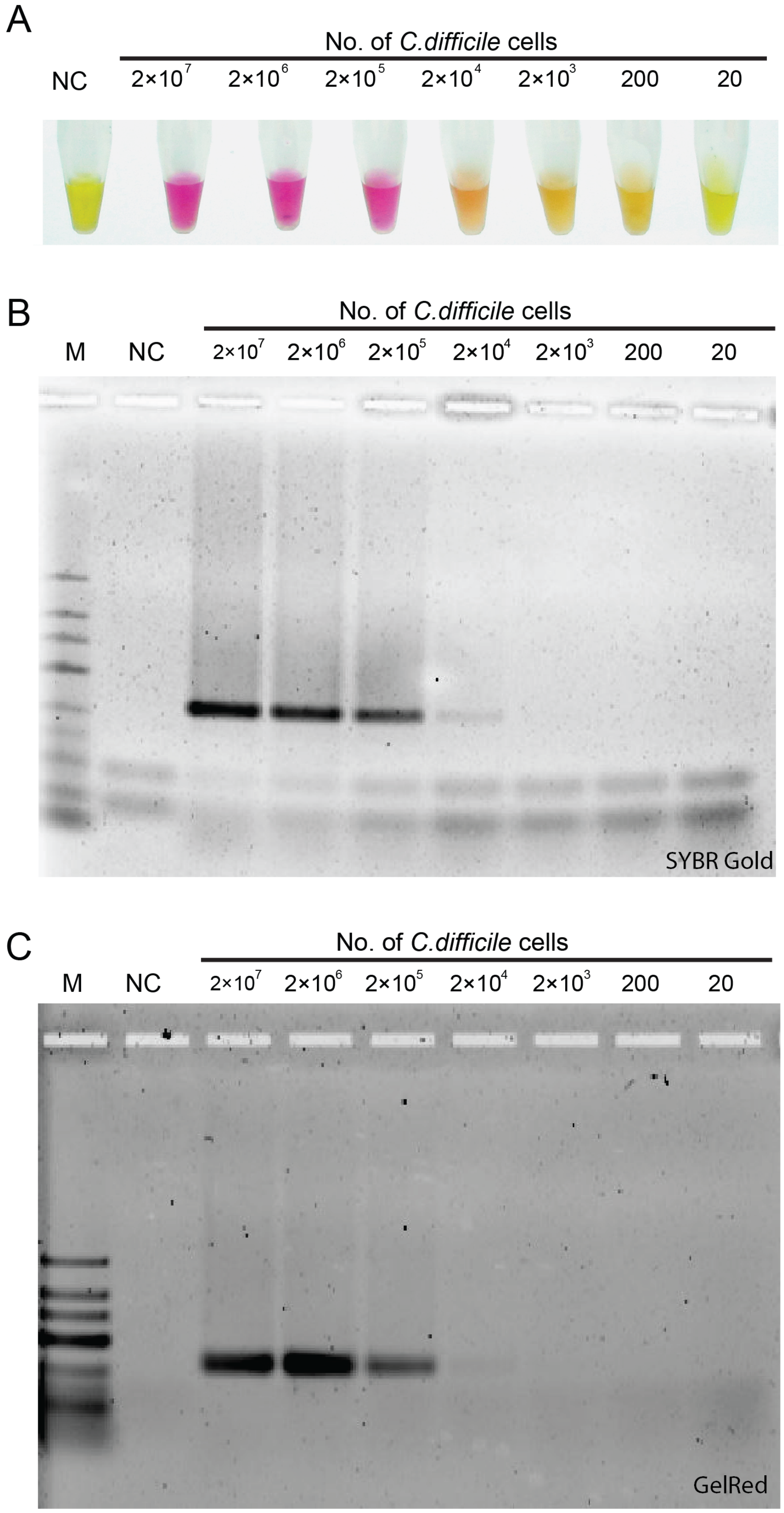

Figure S4. PCR products examined with litmus test (a), 2% agarose gel stained by either SYBR Gold (b) or GelRed (c). Genomic DNA was prepared from the specified number of *C. difficile* cells and subjected to 28 cycles of PCR. The PCR products were then used for each test.

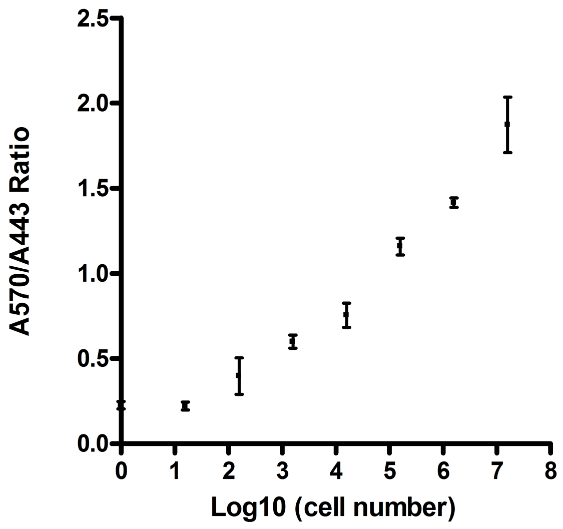

Figure S5. Quantification of PCR-litmus test. Genomic DNA was prepared from the specified number of *C. difficile* cells and subjected to 28 cycles of PCR. The PCR products were then used for the litmus reaction for 1 hour. The absorbance of the reaction at 570 nm and 443 nm (A570 and A443) was measured. A570/A443 was plotted against cell numbers at logarithmic scale.

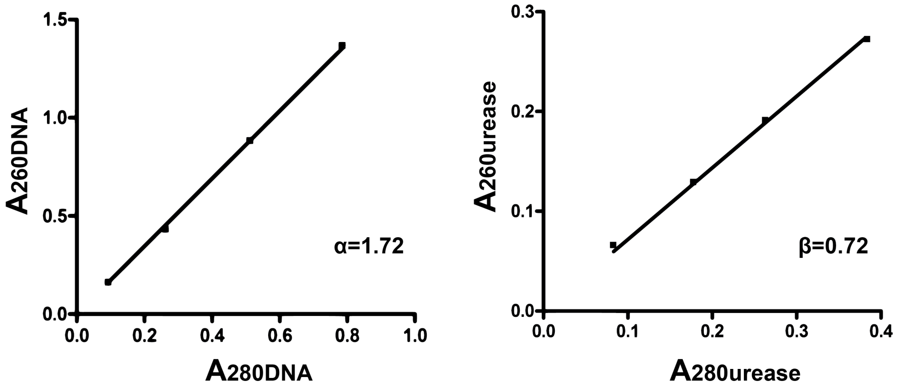

Figure S6. Determination of ratio α and β of the absorbance at 260 and 280 nm of NH_2-DNA and urease.
